# Author Correction: Photonic Generation of High Power, Ultrastable Microwave Signals by Vernier Effect in a Femtosecond Laser Frequency Comb

**DOI:** 10.1038/s41598-018-23234-4

**Published:** 2018-03-14

**Authors:** Khaldoun Saleh, Jacques Millo, Baptiste Marechal, Benoît Dubois, Ahmed Bakir, Alexandre Didier, Clément Lacroûte, Yann Kersalé

**Affiliations:** FEMTO-ST, Univ. Bourgogne Franche-Comté, CNRS, ENSMM, 26 Rue de l’Épitaphe, 25030 Besançon, France

Correction to: *Scientific Reports* 10.1038/s41598-018-20408-y, published online 31 January 2018

The original version of this Article contained a typographical error in the spelling of the author Clément Lacroûte, which was incorrectly given as Clément Lâcroute. This has now been corrected in the PDF and HTML versions of the Article.

